# MSRE-HTPrimer: a high-throughput and genome-wide primer design pipeline optimized for epigenetic research

**DOI:** 10.1186/s13148-016-0190-9

**Published:** 2016-03-05

**Authors:** Ram Vinay Pandey, Pulverer Walter, Rainer Kallmeyer, Gabriel Beikircher, Stephan Pabinger, Albert Kriegner, Andreas Weinhäusel

**Affiliations:** Health & Environment Department, Molecular Diagnostics, AIT—Austrian Institute of Technology GmbH, Vienna, Austria; Institut für Populationsgenetik, Vetmeduni Vienna, Veterinärplatz 1, 1210 Vienna, Austria

**Keywords:** PCR, Primer design, DNA methylation, Methylation sensitive restriction enzyme, High-throughput, CpG islands, MSRE-PCR

## Abstract

**Background:**

Methylation-sensitive restriction enzymes—polymerase chain reaction (MSRE-PCR) has been used in epigenetic research to identify genome-wide and gene-specific DNA methylation. Currently, epigenome-wide discovery studies provide many candidate regions for which the MSREqPCR approach can be very effective to confirm the findings. MSREqPCR provides high multiplexing capabilities also when starting with limited amount of DNA-like cfDNA to validate many targets in a time- and cost-effective manner. Multiplex design is challenging and cumbersome to define specific primers in an effective manner, and no suitable software tools are freely available for high-throughput primer design in a time-effective manner and to automatically annotate the resulting primers with known SNPs, CpG, repeats, and RefSeq genes. Therefore a robust, powerful, high-throughput, optimized, and methylation-specific primer design tool with great accuracy will be very useful.

**Results:**

We have developed a novel pipeline, called MSRE-HTPrimer, to design MSRE-PCR and genomic PCR primers pairs in a very efficient manner and with high success rate. First, our pipeline designs all possible PCR primer pairs and oligos, followed by filtering for SNPs loci and repeat regions. Next, each primer pair is annotated with the number of cut sites in primers and amplicons, upstream and downstream genes, and CpG islands loci. Finally, MSRE-HTPrimer selects resulting primer pairs for all target sequences based on a custom quality matrix defined by the user. MSRE-HTPrimer produces a table for all resulting primer pairs as well as a custom track in GTF file format for each target sequence to visualize it in UCSC genome browser.

**Conclusions:**

MSRE-HTPrimer, based on Primer3, is a high-throughput pipeline and has no limitation on the number and size of target sequences for primer design and provides full flexibility to customize it for specific requirements. It is a standalone web-based pipeline, which is fully configured within a virtual machine and thus can be readily used without any configuration. We have experimentally validated primer pairs designed by our pipeline and shown a very high success rate of primer pairs: out of 190 primer pairs, 71 % could be successfully validated. The MSRE-HTPrimer software is freely available from http://sourceforge.net/p/msrehtprimer/wiki/Virtual_Machine/ as a virtual machine.

**Electronic supplementary material:**

The online version of this article (doi:10.1186/s13148-016-0190-9) contains supplementary material, which is available to authorized users.

## Background

DNA methylation is a chemically stable key player in epigenetics and heritable over many generations of cell divisions [1]. It is the only known endogenous modification of DNA in mammals and refers to the enzymatic, post synthetic addition of a methyl group to the carbon 5 position of the cytosine ring [2]. Bisulfite-based methods and methylation-sensitive restriction enzyme-based PCR (MSREqPCR) methods have been used for detection of DNA methylation. In bisulfite-based methods, the genomic DNA is first treated with bisulfite, thus converting non-methylated cytosine to uracil by deamination while methylated cytosine is protected. However, this procedure has several limitations, including the inability to discriminate between 5-methylcytosine and 5-hydroxymethylcyosine, the degradation of DNA during bisulfite treatment, high experimental time, and the possibility of incomplete conversion under not ideal reaction parameters (Table 1).

**Table 1:** Comparison of different DNA methylation analysis methods

Method	Advantages	Limitations
BSP/MSP	Highly sensitive.	Gives rise to false positivity if bisulfite modification is incomplete.
	Highly specific for particular CpG sites.	Poor design of primers can give rise to inconclusive results.
	Facilitates the analysis of clinical samples with low levels of methylated sequences.	Low multiplexing capabilities.
	Obviates the use of restriction enzymes and eliminates the problem of incomplete enzyme digestion.	Need for methylation specific probes in addition to primers—when conducting BSP and hybridization probe-based distinction of ME/UM sequences.
Bisulfite sequencing	Highly specific. Usually depending on BSP or MSP before sequencing.	Technically demanding; similar to MSP/BSP because depending on BSP amplification.
	Delineates the methylation status of each individual CpG site.	Time consuming and labor intensive.
	Requires very low DNA amounts.	Gives rise to false positivity if bisulfite modification is incomplete.
	NGS-based high multiplexing capability when pooling single-MSP-derived amplicons.	
MSRE	High sensitivity and enables high multiplexing and quantitative read out.	Limited to target regions, which are covered by MSRE recognition sequence.
	Detection of low fraction (0.1-x%) of methylated in unmethylated DNA background.	Gives rise to false positivity if enzyme digestion is incomplete.
	Suitable for multiplexed analyses 50–100 amplicons starting from ng-amounts (e.g., cfDNA).	Internal standards recommended because no direct distinction of ME/UM alleles is feasible based on the sequence (like C vs T upon BS-based analysis in sequences) when aiming NGS-based readout.

MSREqPCR in contrast to bisulfite PCR can be used for the rapid, simultaneous detection of DNA methylation in multiple fragments when only a limited amount of DNA is available. It is a procedure based on the fact that digestion of genomic DNA with methylation-sensitive restriction enzymes is blocked when methylated. Best suited for that analyses targeting 5-methylcytosine are enzymes, which contain CpG motifs in their recognition sequence such as *AciI*, *Hin6I*, *HpaII*, and *HpyCH4IV* [3, 4]. MSREqPCR-based method allows for a high level of multiplexing with manageable efforts regarding assay optimization, and only a few nanograms of DNA (10–20 ng) are needed per 100 assays [5]. However, this method has some limitations such as (1) all CpGs without having the cut site for MSRE cannot be analyzed and (2) single C-resolution is not feasible for the assays with more than one cut sites in CpG and one has to assume that investigated regions are homogenously methylated, which is a common assumption in epigenetics [6]. However, using different combinations of MSREs and varying product length can adjust the number of CpGs per assay (Table 2). A restriction map can help to identify the correct MSRE (or any combination of different MSREs) and gives an overview where and how often the fragments of interest get cleaved (Table 2). In the present study, to maximize the number of CpGs covered by MSREs, a combination of four different enzymes was chosen to conduct the experiment. The total percent coverage of CpGs for these four enzymes is about 39 % (*AciI*, 17.4 %; *Hin6I,* 6.4 %; *HpaII*, 8.6 %; and *HpyCH4IV,* 6.6 %) of the whole human genomic DNA (Table 2) [4].

**Table 2:** Whole genome MSREs coverage of CpGs in human genomic DNA [4]

MSRE	Recognition sequence	Percentage coverage of CpGs in human gDNA (%)	Number of fragments (per kb) in CpG islands	Number of fragments (per kb) in non-CpG islands
HpaII	C^CGG	8.6	3.98	1.18
Hin6I	G^CGC	6.4	3.98	0.61
AciI	C^CGC	17.4	3.23	1.79
HpyCh4IV	A^CGT	6.6	1.31	1.08
Bsu15I	AT^CGAT	0.2	<0.01	0.02
NarI	GG^CGCC	0.6	1.08	<0.01
Bsp119I	TT^CGAA	0.1	0.11	<0.01
Psp1406I	AA^CGTT	0.3	<0.01	0.05
XmiI	GT^MKAC	0.1	0.19	0.34
Hin1I	GR^CGYC	2	1.92	0.11

In past years, clinical epigenetic studies have shown the importance and successful utilization of the MSRE-PCR method [3–5, 7–11]. Despite the successful use of this method, there are no one-stop software tools available to design a large number of optimized MSRE-specific assays in a time-efficient manner in parallel. There are several software tools available to design general-purpose PCR primer pairs as well as oligos such as PerlPrimer [12], Primer3 [13], Primer3Plus [14], Primer Express [15], Primer Select [16], BatchPrimer3 [17], Primer Premier [18], PRIMEGENS [19], and PrimerBlast [20]. These tools are not optimized for methylation-specific primer design and do not support multiplexed and high-throughput primer design in a time-effective manner.

Primer3 is a widely used program for designing PCR primers and is the basis for many other tools, but does not provide genomic information about resulting primer pairs and only reports relative coordinate information to the target sequence. BatchPrimer3 is a batch primer tool, which allows use of multiple target sequences but has limitations on target length and does not design MSRE-PCR primers. None of the other tools provide MSRE-PCR design, genomic annotation (i.e., single-nucleotide polymorphism (SNP), CpG locus annotation, upstream and downstream RefSeq gene information and cut site information), and efficient and accurate primer pair selection by using a user-defined quality-filtering matrix to reduce the post processing.

PerlPrimer and PRIMEGENS perform DNA methylation-based primer design by identifying the cut sites and CpG islands, but they do not provide genome, SNP, and repeat information for filtering primer pairs. Furthermore, they do not support user-defined primer pair selection and filtering to pick the best and reliable primer pairs.

We have therefore developed MSRE-HTPrimer, an open source, web-based, and high-throughput primer design pipeline for MSRE-PCR and genomic-PCR primers capable of simultaneously processing hundreds to thousands of target sequences. To achieve that goal, we have adapted the current Primer3 primer design process and added genomic annotations, multiprocessing capabilities, and new primer selection possibilities. The setup and validation of MSREqPCR were done in accordance with the MIQE guidelines, representing the minimum information for publication of quantitative real-time PCR experiments [21] to ensure reliable experimental results.

## Results and discussion

**Fig. 1:**
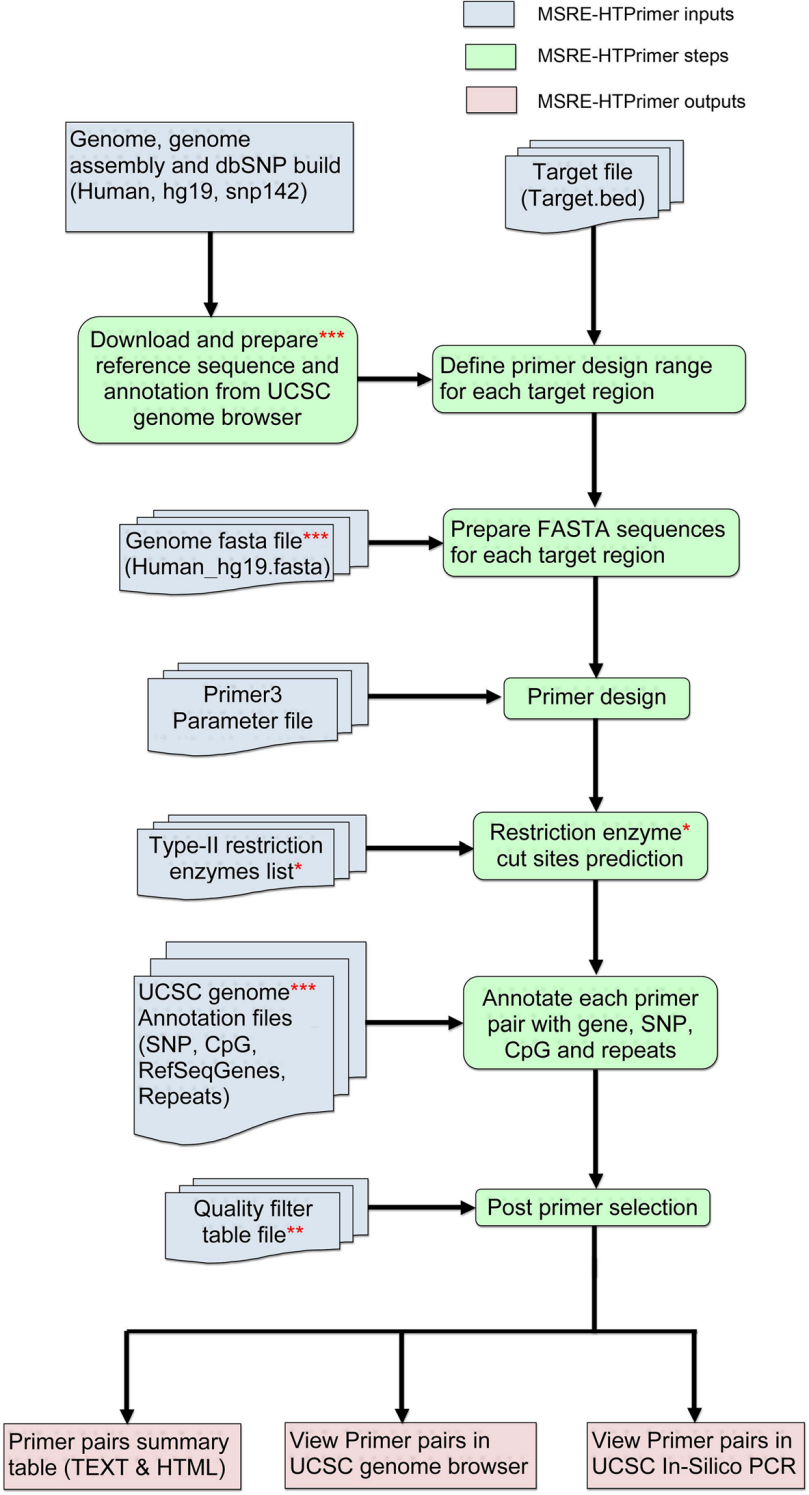
The workflow of MSRE-HTPrimer pipeline. The MSRE-HTPrimer pipeline can be run via an intuitive web interface. The sequential analysis steps are displayed from *top* to *bottom*. (*Single asterisk*) *Restriction enzyme cut sites prediction* step and the *Type-II restriction enzymes list* input are only applicable for MSRE-PCR. (*Double asterisks*) Quality filter table file is optional; if user-defined primer selection criteria are not provided, then all primer pairs will be recorded in the final output summary table. (*Triple asterisks*) Download and prepare reference sequence and annotation from UCSC genome browser step runs only once first time or when reference genome information is changed

MSRE-HTPrimer is an open-source, portable, web-based, and easy-to-use pipeline, which facilitates the design of primer pairs for epigenetic and genomic target validation studies. It uses a simple input and output model and can design primers for hundreds to thousands of target sequences in a single run. Moreover, it does not have any limitations on the number and size of target sequences. MSRE-HTPrimer provides significant improvements over existing solutions with following unique features: (1) visualization of primer pairs in UCSC genome browser [22], (2) search each resulting primer pair in UCSC In-Silico PCR database [23], (3) flexible primer selection and filtering based on custom quality matrix, and (4) parallel primer design for several target sequences. The pipeline is equipped with multiprocessing capability and uses custom inputs and parameters to design specific primers. All components of MSRE-HTPrimer pipeline workflow, inputs, and outputs have been summarized in Fig. 1.

### MSRE-HTPrimer pipeline

The MSRE-HTPrimer pipeline (Fig. 1) consists of seven sequential steps. Based on a user-defined list of target regions and design parameters, the pipeline retrieves a list of annotated primer pairs (in TEXT and HTML format) and links to visualize results in UCSC genome browser and UCSC In-Silico PCR. All steps of the pipeline are described as following:

1. Download and prepare reference sequence and annotation from UCSC genome browser
  After installation, when the user starts the first primer design process, MSRE-HTPrimer downloads and prepares the reference FASTA sequence, common SNPs, RefSeq gene, CpG islands, and known repeat elements annotation for the entire genome (human and mouse) based on the selected genome, genome assembly, and dbSNP build number from the UCSC genome browser. The default genome on query page is human, genome assembly is hg19, and the dbSNP build number is 142. MSRE-HTPrimer does not re-download reference data for subsequent primer design if it is already downloaded and prepared. From the query interface, the user can customize all primer design and selection parameters for both MSRE-PCR and genomic-PCR.
2. Define primer design range for each target region
  In this step, the primer design genomic range is prepared by adding the number of flanking upstream and downstream base pairs (optional) to the actual target region given as input in the target bed file (Additional file 1).
3. Prepare FASTA sequence for each target region
  Next, target FASTA sequences are extracting from the genome reference using the target regions from step 2. The genome reference FASTA file and common SNPs, RefSeq gene, CpG islands, and “repeats-annotation” files for human or mouse are downloaded and prepared from the UCSC genome browser in step 1. In this step, MSRE-HTPrimer also subset the large annotation files based on target regions coordinates using BedTools [24], which makes execution faster.
4. Primer design with Primer3
  In this step, the tool takes two inputs: (1) FASTA sequences from the previous step and (2) a Primer3 parameter input file (Additional file 2). It runs the Primer3 tool [25] to design all possible PCR primer pairs and oligos (optional) for all targets sequences and stores all resulting primers, oligos, and amplicons.
5. Restriction enzyme cut sites prediction
  In this step, the enzymes cut sites in forward primers, reverse primers, oligos, and amplicons are calculated. This step is only applicable for MSRE-PCR primer design; optionally, when users design genomic PCR primers, this step can be omitted.
6. Annotate each primer pair with gene, SNP, CpG, RefSeq genes, and repeats
  In this step, each primer pair, oligo, and amplicon is annotated with RefSeq genes found upstream and downstream of the target region, SNPs, CpG islands, and repeat regions. These annotations will help to pick the accurate and sensitive primer pairs for each target region.
7. Post primer selection

Based on the user-defined selection criteria, the final primer pairs for each target region are selected. These selection criteria can be given as and input file which is specified in Additional file 3. This step facilitates selection of primer candidates and provides the specific primer pairs for hundreds to thousands of target regions in a time-effective manner. This hierarchical filtering process is a unique and very useful feature of the MSRE-HTPrimer, which is lacking in all other similar available tools. Finally, MSRE-HTPrimer produces primer summary table in TEXT and HTML format and visualizes results in UCSC genome browser [22] and UCSC In-Silico PCR database [23].

### Query interface

**Fig. 2:**
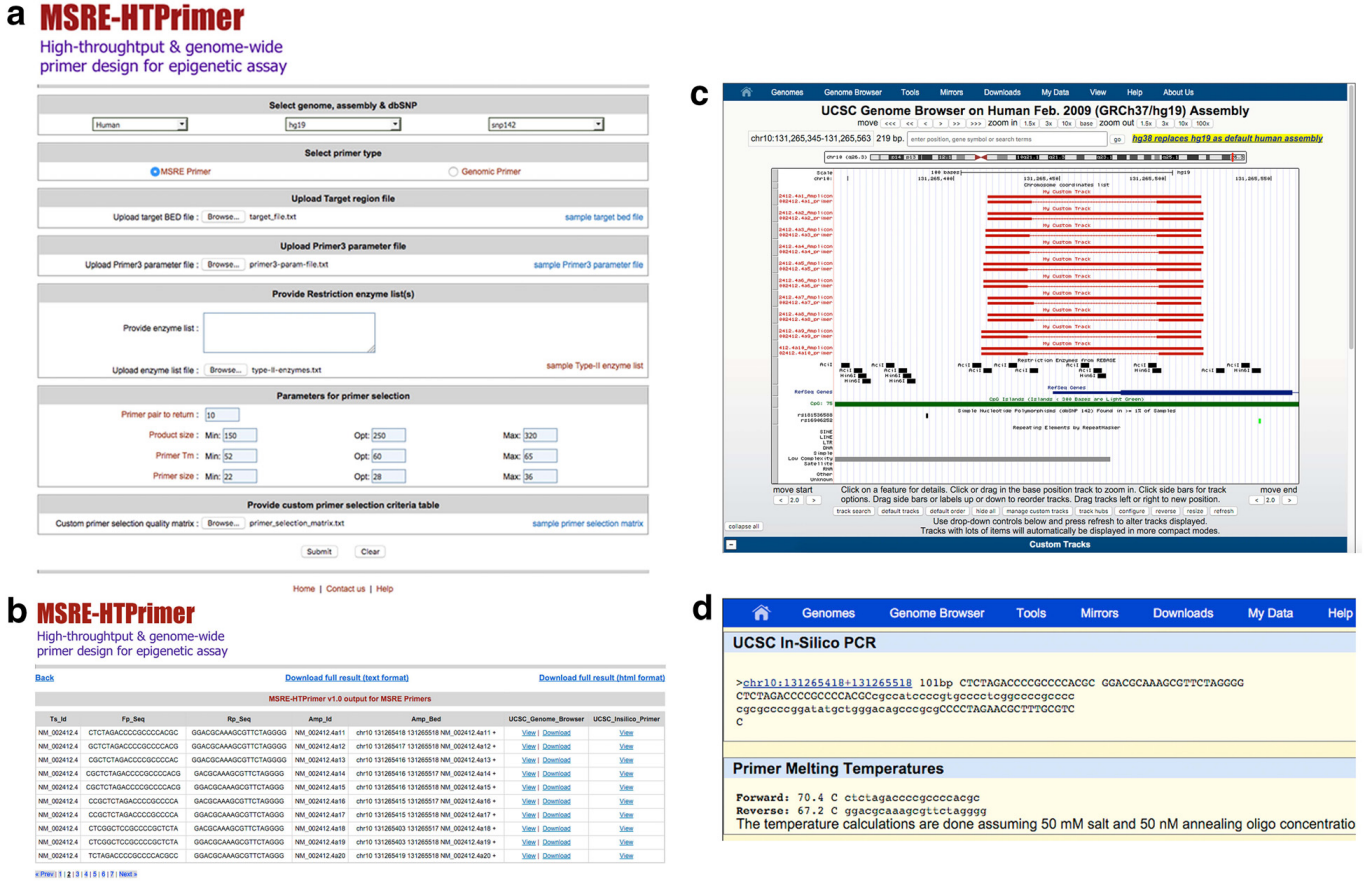
The MSRE-HTPrimer web interface and output. **a** Web interface of MSRE-HTPrimer query page (the query interface for MSRE-HTPrimer shows different parameters that can be used to design and select optimized PCR primers for MSRE assay and genomic sequencing assay). **b** An example of primer pair summary output table in MSRE-HTPrimer web interface (an example of primer pair summary table for RefSeq Human gene MGMT [*chr10: 129467190–129768042, RefSeq ID NM_002412.4]*. It contains target sequence ID, forward and reverse primer sequence, amplicon coordinates in BED format, link to display and download to UCSC genome browser, and display to In-Silico PCR database). **c** An example output of the MSRE-HTPrimer primer pair visualization in UCSC genome browser along with genomic (MSRE cut sites, RefSeq, CpG islands, SNPs, and Repeats) track within MSRE-HTPrimer web interface (in order to focus and illustrate MSRE-HTPrimer features, all redundant UCSC genome browser tracks are hidden in 2C presenting from *top* to *bottom*; the MSRE cutsites position, the localization of RefSeq Human gene MGMT [*chr10: 129467190–129768042, RefSeq ID NM_002412.4]*, CpG islands, simple nucleotide polymorphisms based on dbSNP 142 [rs18153536588 and rs16906252], and the kind of repeating elements [low complexity]. Each result of the primer design pipeline is presented bundled, once as *single red line* [full amplicon] and as a line emphasizing forward and reverse primer below). **d** An example output of In-Silico PCR database search display of primer pairs (an example of primer pair display in UCSC In-Silico PCR database for RefSeq human gene MGMT [*chr10: 129467190–129768042, RefSeq ID NM_002412.4])*

MSRE-HTPrimer offers a very intuitive, user-friendly, and powerful query interface (Fig. 2a). MSRE-HTPrimer allows the user to design genome-wide primers for any number of target sequence in a single run. The user can select the appropriate genome name, genome assembly, and the dbSNP build as per requirements. For primer design, the user can upload a target bed file, Primer3 parameter file, type-II enzyme list for MSRE-PCR primer, and custom primer selection matrix file. Moreover, the user can customize several primer design and selection parameters to obtain specific and optimized primer pairs and product. The query page can be opened with http://localhost/msre-htprimer page.

### MSRE-HTPrimer input

MSRE-HTPrimer requires four input files:

1. Target BED file: This file contains the genomic coordinates for all target sequences (one line for each target sequence). It consists of four tab-delimited columns: (1) chromosome, (2) start coordinate, (3) end coordinate, and (4) a unique ID for each target region (Additional file 1).
2. Primer3 parameter file: This text file contains the parameters and values for the Primer3 tool. It is optional and if not provided, MSRE-HTPrimer will use default Primer3 parameters (Additional file 2)
3. Restriction enzyme file: This input file is only required for MSRE-PCR primer design. Each line contains an enzyme name as per nomenclature, and multiple enzymes are allowed in a single run (Additional file 4).
4. Custom primer selection quality matrix.

MSRE-HTPrimer supports selection of primer pairs based on user-defined selection criteria. A custom quality-filtering matrix can be provided as input file. As shown in Additional file 3, the user can define a set of selection criteria and rank them using a scale of 1–10. MSRE-HTPrimer assigns these ranks to the primer pairs for all target sequences. If this input is not provided, then primer pairs are returned based on Primer3 ranking. MSRE-HTPrimer supports mathematical operators, including “>,” “<,” “>=,” “<=,” and “-“. Any column header of the MSRE-HTPrimer output file can be used as parameter. The primer quality level represents the hierarchical rank associated with each of the output parameters in its respective row.

### MSRE-HTPrimer output

MSRE-HTPrimer produces a summary output file in two formats: (1) a tab delimited text file and (2) HTML output file (See Additional file 5 and Fig. 2b), which contains one line for each primer pair along with all annotations including target sequence ID, amplicon ID, oligos and genome amplicon coordinates, number of cut sites, number of SNPs, number of CpG islands, repeat regions, upstream and downstream RefSeq genes and their distance with respect to forward and reverse primer, and direct link to UCSC genome browser and UCSC Insilco-PCR. For both primer design methods (MSRE-PCR and Genomic-PCR), it produces a uniform output, which facilitates an easy output handling, post processing, and management. The HTML summary output table has a direct hyperlink to UCSC genome browser and UCSC In-Silico PCR database.

### Visualization of primer pairs

MSRE-HTPrimer offers the visual display of resulting primer pairs in UCSC genome browser along with genomic annotations, CpG islands, common SNPs, RefSeq genes, restriction enzymes and other genomic information (Fig. 2c), and in UCSC In-Silico PCR database (Fig. 2d). In addition, MSRE-HTPrimer also provides the primer pairs for each target sequence as a UCSC custom track file in GTF format (Additional file 6), which can be used for other analysis or can be visualized in other genome browser. The custom track bed file is created for each target sequence and can be downloaded from the summary output table (see Fig. 2b).

### Availability, installation, and usage

MSRE-HTPrimer is a stand alone, portable, and web-based pipeline, freely available for all researchers. It is available as a fully configured Virtual Machine accessible at http://sourceforge.net/p/msrehtprimer/wiki/Virtual_Machine/. An extensive user manual is available at https://sourceforge.net/projects/msrehtprimer/files/Manual.pdf. A test data set is available at https://sourceforge.net/projects/msrehtprimer/files/test_data.zip.

### Performance evaluation

**Fig. 3:**
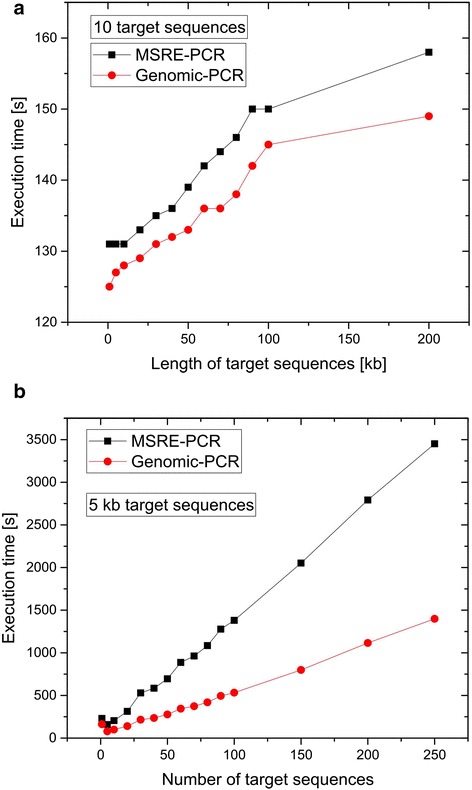
Evaluation of MSRE-HTPrimer execution for MSRE-PCR (*black line*) and genomic-PCR (*red line*), considering the number and size of target sequences. **a** Different sizes of target sequences (1, 5, 10, 20, 30, 40, 50, 60, 70, 80, 90, 100, and 200 KB), each time using 10 target sequences of the same length. **b** Different numbers of target sequences (1, 5, 10, 20, 30, 40, 50, 60, 70, 80, 90, 100, 150, 200, and 250 sequences of equal length of 5 kb)

MSRE-HTPrimer is a high-throughput primer design pipeline and has no restriction either on the number of assays or on the size of target sequences. To evaluate the performance of MSRE-HTPrimer, we have randomly selected 200 target sequences of different lengths from human gene ESR1-estrogen receptor 1 (chr6:152011631–152424408) and executed the benchmarking on a Linux server (Ubuntu 12.0.4 LTS with 8 CPU, 16 GB RAM). Execution times were measured for both MSRE-PCR (black line) and genomic-PCR (red line). All benchmark measurements have been performed using the Primer3 version 2.3.6 focusing on different sizes and number of target sequences in relation to runtime. All execution times were measured in seconds. As shown in Fig. 3a, b, MSRE-HTPrimer is very fast and efficient to design specific primer pairs for hundreds of target regions. As shown, design for 100 MSRE-PCR assays is conducted in less than 1500 s (25 min) computing time to run the entire steps according to the pipeline. For the same dataset, MSRE-PCR design takes more time than genomic-PCR design (e.g., <500 s designing 100 assays), which is due to restriction enzyme cut site annotation (Fig. 3b).

### MSRE-HTPrimer experimental validation

**Fig. 4:**
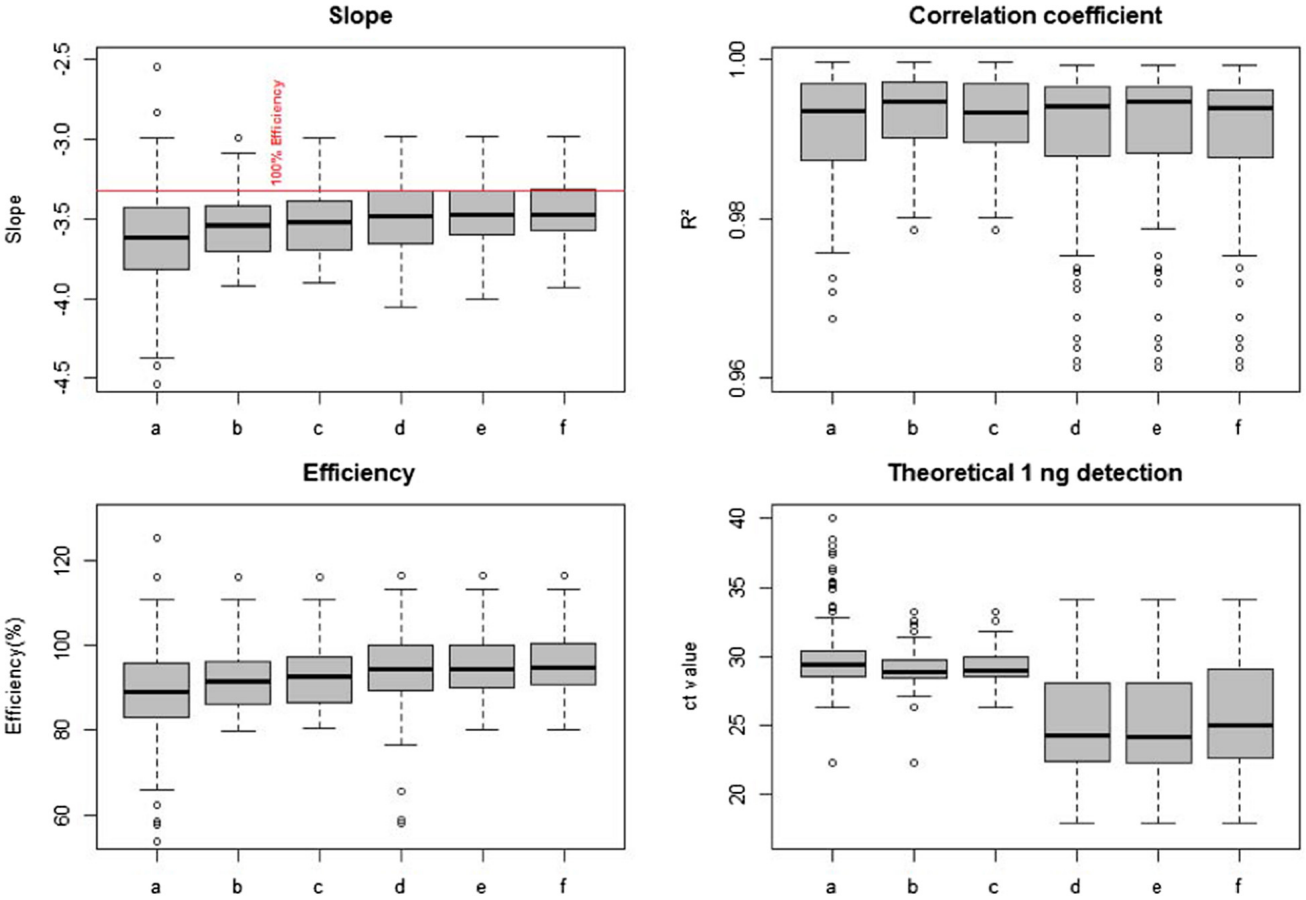
qPCR-based evaluation of the performance parameters of designed assays. The *boxplots* illustrate the slope, correlation coefficient, qPCR efficiency, and the theoretical 1 ng detection of singleplex and multiplex qPCR experiments on Roches Lightcycler 480. *Columns a*, *b*, and *c* show *boxplots* from the singleplex experiments including all 200 tested assays (*a*), successfully tested assays in singleplex reactions (135, *b*), and final selected 90 MSREqPCR assays (*c*). *Columns d*, *e*, and *f* show the multiplex results including 135 tested assays (*d*), successful tested assays in multiplex reactions (128, *e*), and again the performance of final chosen 90 assays (*f*)

We have experimentally validated the primers designed by our MSRE-HTPrimer pipeline for methylation sites selected from a genome-wide discovery study. Predefined design and filtering parameters of the MSRE-HTPrimer tool, such as the amount of cut sites per assay, position of the cut sites, SNP filtering, and avoiding position with repeats, combined with the standard parameters of Primer3 (e.g., sequence length, melting temperature, GC content, and primer length) yielded a total of 190 MSREqPCR assays. Running those under the same PCR conditions, out of 190 primer pairs, 135 (71.05 %) were qualified for qPCR according MIQE guidelines. Details on performance parameters can be found in the Additional file 7. The validation results are shown in Fig. 4and include the performance parameters *efficiency*(deduced from *slope* by the formula *E* = (10^(−1/slope)^ − 1)*100; average efficiency of 135 assays, 92.03 %), the *correlation coefficient*(average *R*^2^, 0.993) and the *theoretical 1 ng detection*(average ct: 29.11) of the assays, which were deduced from the calibration curve. These results are in line with our experiences when qualifying “manually” designed sets of assays and also comparable to MSP as well as BSP-based assays where our internal success rate is between 70 and 80 % (data not shown).

### Intended user groups

The major advantage of the MSRE-HTPrimer pipeline in contrast to other bioinformatics primer design tools is its flexibility and multiprocessing capability to design primers for thousands of targets in parallel. Moreover, it decreases the post-processing steps by applying efficient filtering and providing selection criteria based on a user-defined quality-filtering matrix. It allows users to develop optimized assays and thus significantly increases speed and success rate of genetic as well as epigenetic primers design for validation. The indented user group includes researchers working in the genetic and epigenetic domain. The genomic-PCR method of MSRE-HTPrimer adds another level of information to the primer design by giving researchers the possibility to design primer pairs based on specific genomic traits or locations (SNP, RefSeq, CpG islands, repeats). Furthermore, the MSRE design approach enables the design of primer pairs specifically for the analysis of DNA methylation or for bisulfite deamination-based MSP.

### Comparison with existing tools

Today, several open source and commercial primer design software/tools are available including, PerlPrimer, Primer3, Primer3Plus, PrimerSelect, Batchprimer3, PrimerPremier, PRIMEGENS, and PrimerBlast. The comparison of the MSRE-HTPrimer pipeline to each of these tools is shown in Table 3. In total, 19 different points including availability, operating system, and installation requirements as well as necessary dependencies, multiprocessing capabilities, limitations of input file size, target sequence length, amount of target sequences, visualization of results in UCSC genome browser, and genomic annotation of results were evaluated. MSRE-HTPrimer is available for free and can be run on any Unix system. Furthermore, it is available as a fully configured virtual machine. MSRE-HTPrimer has no limitations concerning the input file size, the amount of target sequences, or the target sequence length. Moreover, it is capable of multiprocessing the target sequences reducing the time for high-throughput primer design. In addition, MSRE-HTPrimer provides many advance features over existing tools such as (1) assigning genomic coordinates to all resulting primer pairs, oligos, and amplicons; (2) annotate all resulting primers with SNPs, RefSeq genes, repeat elements and CpG islands and automatic FASTA sequence preparation based on input target BED file (Additional file 1); (3) positioning of restriction enzyme cut sites accurately; and (4) visualization of results in the, e.g., UCSC genome browser. Finally, MSRE-HTPrimer is extremely useful for methylation-sensitive restriction enzyme-based PCR and any genetic primer design using non-bisulfite deaminated DNA sequences.

**Table 3:** Comparison of various features of MSRE-HTPrimer and other tools for primer design

Features	MSRE-HTPrimer v1.0	Primer Express v3.0.1	Primer Select	Primer Premier	Perl Primer v1.1.21	PRIMEGENS-v2.0	BiSearch
Genome-wide primer design	Yes	No	No	Yes	No	No	No
Dependency	No	Yes	Yes	Yes	Yes	Yes	Yes
Installation required	No	Yes	Yes	Yes	Yes	Yes	No
Operating system	All Unix, MAC, Windows	Windows	–	Windows, MAC	All Unix, MAC, Windows	All Unix, Windows	Web
Genome coordinate information^a^	Yes	No	No	No	No	No	No
SNP annotation^a^	Yes	No	No	Yes	No	No	No
Repeat element annotation^a^	Yes	No	No	Yes	No	No	No
CpG Islands annotation^a^	Yes	No	No	No	Yes	No	No
RefSeq gene annotation^a^	Yes	No	No	Yes	No	No	No
Restriction enzyme type-II cut sites identification^a^	Yes	No	No	No	No	No	No
Multi-processing capability	Yes	Yes	No	Yes	No	Yes	No
Multiple target sequences	Yes	Yes	No	Yes	No	Yes	No
Target sequence number restriction^a^	No	Yes	Yes	Yes	Yes	Yes	Yes
Target sequence length restriction^a^	No	Yes	Yes	Yes	Yes	Yes	Yes
FASTA sequence selection by tool	Yes	No	No	Yes	Yes	No	No
Custom primer selection quality matrix^a^	Yes	No	No	No	No	No	No
Input file limitations	No	No	No	No	No	No	No
UCSC genome browser visualization^a^	Yes	No	No	No	No	No	No
UCSC In-Silico primer design page cross-link	Yes	No	No	No	No	No	No
Availability	Free	Commercial	Commercial	Commercial	Free	Free	Free

^a^Denotes the unique feature of MSRE-HTPrimer in comparison to the other tools

## Conclusions

We provide MSRE-HTPrimer, a robust, user-friendly, web-based, standalone, one-stop high-throughput, and genome-wide epigenetic primer design pipeline with multiprocessing capabilities. MSRE-HTPrimer annotates all resulting primer pairs extensively by adding genetic and epigenetic information including SNPs, RefSeq genes, repeats, and CpG islands. It enables primer design for thousands of target sequences in a single run with great accuracy and greatly facilitates post processing. MSRE-HTPrimer has no limitation on the number and size of target sequences and provides full flexibility to customize the Primer3 parameters for each specific requirement. Furthermore, it offers the opportunity to rank primer pairs based on task-specific preferences using a custom quality filter matrix in addition to general Primer3 ranking. In comparison to other tools, MSRE-HTPrimer stands out for high-throughput, genome-wide, and optimized epigenetic primer design capability, improved primer design accuracy, efficient primer selection, and primer visualization in UCSC genome browser.

## Methods

### Pipeline development

The MSRE-HTPrimer pipeline was developed using Python 2.7.10 (http://www.python.org) and Biopython (http://biopython.org) with special focus on multiprocessing capability to design two types of primers: (1) epigenetic primers (MSRE-PCR) and (2) genomic and sequencing primers (genomic-PCR) with great efficiency and success rate in a high-throughput manner. The reference genome FASTA sequence and annotations are used from UCSC genome browser, which are automatically retrieved and prepared by MSRE-HTPrimer. The minimum user-defined input requirements to run MSRE-HTPrimer are (1) target genomic regions in BED format file, (2) type-II enzyme(s) for MSRE primers, (3) genome name, assembly, and dbSNP version. The MSRE-HTPrimer workflow is depicted in Fig. 1 consisting of seven sequential steps and starts with an arbitrary list of target regions and outputs a list of annotated primer pairs.

### Web interface development

The MSRE-HTPrimer web interface was developed using the HTML, Perl, and CGI and runs on an Apache web server. The graphical display of designed primer pairs and products for all target sequences are visualized in the UCSC genome browser and the UCSC In-Silico PCR database. Hence, MSRE-HTPrimer uniquely depicts the designed primer pairs in the UCSC genome browser along with genomic annotation, restriction enzymes, repeats, conservation, RefSeq genes, and other information available in UCSC genome browser.

An extensive user manual (Additional file 8) is provided including description of inputs, parameters, outputs, installation dependencies, MSRE-HTPrimer usage, and a detailed step-by-step description of the MSRE-HTPrimer pipeline. The tool along with intuitive web interface is available as a fully configured Virtual Machine (VM), which can be run using the Virtual box system (https://www.virtualbox.org/). The virtual machine is configured to run without any installation and configuration needs.

### Experimental validation of MSRE-HTPrimer

An epigenome-wide experiment using DNA methylation arrays from Illumina (Infinium HumanMethylation450 Bead Chips, Illumina, California, USA) was used to define regions for assay design and to further experimentally qualify the PCR primers designed by the MSRE-HTPrimer pipeline in the lab. The Infinium 450k assay provides distinct information about the methylation level of 485,577 cytosine sites per sample at single-nucleotide resolution within the human genome. Based on statistical analysis of the 450k data as well as other “biological” criteria (data not shown), a panel of 190 target regions referring to corresponding CpG sites presented by the 450k array (Additional file 7 and Additional file 9) were selected for methylation analyses by MSRE-PCR. The MSRE-PCR design parameters were set as following: the PCR product must have at least one restriction enzyme cut site ideally within the amplicon as well as no SNP within the primer sequence and no common repeats within the assay. Furthermore, each original target position has to be situated inside or close (±50 bp) to the PCR sequence. MIQE conform performance parameters of the designed assays was evaluated by qPCR using a Roche Light Cycler 480 system. Therefore, DNA from peripheral blood was serially diluted and applied to the qPCR reactions to create a calibration curve with four calibration points (10 ng/reaction; 2.5 ng/reaction; 0.625 ng/reaction; 0.156 ng/reaction—dilution-factor 4).

The performance parameters were directly deduced from the calibration curve: A slope of −3.32 correlates with 100 % qPCR efficiency and indicates a doubling of the strands during PCR. The theoretical 1 ng detection gives information of the expected cp-values when only 1 ng of DNA is applied to the qPCR reaction and is directly deduced from the intersection with the *y*-axis (intercept). Performance of the assays was evaluated in single-plex reactions as well as in multiplex reaction. For the multiplex reaction, a pre-amplification containing all 135 primer pairs was executed on a conventional PCR cycler; subsequently, the pre-amplified targets were analyzed by Roche’s Light Cycler 480.

## Additional files

Additional file 1: Target bed file (mandatory input file) to run primer design with MSRE-HTPrimer. (TXT 256 bytes) Additional file 2: Primer3 parameter input file. This is the parameter for Primer3 tools to design primer pairs. Users can change these parameters to optimize and better positioning of the primer pairs for target region. These parameters are directly supplied to Primer3 tool. (TXT 477 bytes) Additional file 3: Custom quality filter matrix with ten quality levels ranking the designed primer independent of the Primer3 level, but dependent on amplicon size, amount of cut sites, and gene distance. (TXT 870 bytes) Additional file 4:
List of restriction enzymes (mandatory input for MSRE-PCR) in a text file one enzyme per line. (TXT 30 bytes) Additional file 5:
MSRE-HTPrimer output summary table. MSRE-HTPrimer produces a final output summary with one line per primer pairs including target sequence information, primer pairs, oligos, amplicons, and their genomic and epigenetic annotations. (XLS 234 kb) Additional file 6: An example output UCSC custom track in GTF format produced by MSRE-HTPrimer for each target sequence in a separate file. (GTF 3 kb) Additional file 7: Detail description of 135 experimentally validated MSREqPCR assays designed by MSRE-HTPrimer. (XLSX 57 kb) Additional file 8: MSRE-HTPrimer user manual. An extensive guide for user to design primers with MSRE-PCR and genomic-PCR methods. (PDF 7698 kb) Additional file 9: The target bed file, which contains 190 target regions, used for experimental validation. (TXT 7 kb)
