# Erratum to: MSRE-HTPrimer: a high-throughput and genome-wide primer design pipeline optimized for epigenetic research

**DOI:** 10.1186/s13148-016-0250-1

**Published:** 2016-08-04

**Authors:** Ram Vinay Pandey, Walter Pulverer, Rainer Kallmeyer, Gabriel Beikircher, Stephan Pabinger, Albert Kriegner, Andreas Weinhäusel

**Affiliations:** 1 Health & Environment Department, Molecular Diagnostics, AIT—Austrian Institute of Technology GmbH, Vienna, Austria; 2 Institut für Populationsgenetik, Vetmeduni Vienna, Veterinärplatz 1, 1210 Vienna, Austria

## Erratum

Unfortunately, the original version of this article [1] contained an error. The name of one of the authors was included incorrectly and it read “Pulverer Walter” instead of “Walter Pulverer”.

The name has been updated in the original article and is correctly included in full in this erratum.
