# Supplementary material for: MSRE-HTPrimer: a high-throughput and genome-wide primer design pipeline optimized for epigenetic research
Source: Clin Epigenetics. 2016 Mar 5;8:26. doi: 10.1186/s13148-016-0190-9 (PMC4779238; doi:10.1186/s13148-016-0190-9)
Supplement: Additional file 8: — MSRE-HTPrimer user manual. An extensive guide for user to design primers with MSRE-PCR and genomic-PCR methods. (PDF 7698 kb) [file 13148_2016_190_MOESM8_ESM.pdf]

MSRE-HTPrimer User Guide, Version 1.0

Health & Environment Department
AIT Austrian Institute of Technology GmbH
Vienna 1190, Austria
January 26 2016

TABLE OF CONTENTS

1. What is MSRE-HTPrimer? ………………………………………………… 3

2. System requirements …………………………………………………… 3

3. How to obtain MSRE-HTPrimer? ………………………………………..… 4

4. MSRE-HTPrimer web interface description ……………………………..….. 4

4.1 MSRE-HTPrimer query page ……………………………………… 4

4.1 Download MutAid Virtual Machine ………………………………..……. 4

4.1.1 How to use Virtual Machine? ……………………………....... 4

4.2 MSRE-HTPrimer result page ……………………………………… 7

4.3 Run MSRE-HTPrimer with test input ……………………………… 9

5. MSRE-HTPrimer inputs description …………………………...……....... 9

6. How to use MSRE-HTPrimer? ……………..…………..……..…………… 12

7. Contact information …………………………………………………………… 12

1. What is MSRE-HTPrimer?

MSRE-HTPrimer is a high-throughput and genome wide primer design pipeline for epigenetic and genomic sequencing primer design for hundreds to thousands of target sequences in a single run with great accuracy, sensitivity and success rate. It offers easy to handle input and output options, which is especially designed for non-expert users.

MSRE-HTPrimer provides significant improvements over existing solutions with following unique features 1) parallel primer design for several target sequences, 2) primer selection and ranking based on custom filtering criteria, 3) links to Insilico-PCR for each resulting primer pair, 4) visualization of the primer design result in UCSC genome browser, and 5) takes SNPs and repeats into consideration for primer design and automatically discard primer pairs which falls in repeat region or contain SNPs. The pipeline is equipped with an intuitive web interface and multiprocessing capability and provides custom inputs and parameters to design target specific primers. MSRE-HTPrimer is a user-friendly and standalone tool, which is available within a fully configured Virtual machine. It does not require any installation or configuration except VirtualBox (http://www.virtualbox.com).

2. System requirements

Virtual box from https://www.virtualbox.org/

Operating system

Linux

Mac OSX 10.6 or later

Windows PC

3. How to obtain MSRE-HTPrimer?

MSRE-HTPrimer is a web-based standalone tool available within a fully configured Virtual box and can be downloaded from https://sourceforge.net/p/msrehtprimer/wiki/Virtual_Machine and can be easily run on any operating system. With Virtual machine no installation and configuration required.

Once virtual machine of MSRE-HTPrimer is obtained then follow these steps to run the MSRE-HTPrimer tool.

Step1: Download and install Virtual Box from https://www.virtualbox.org/

Step2: Import MSRE-HTPrimer Virtual Machine file into Virtual Box

Step3: Login into MSRE-HTPrimer Virtual machine with username = **testuser** and password = **testuser**

Step4: Open Firefox or any other web browser and open the query page of MSRE-HTPrimer with http://localhost/msre-htprimer

Step5: Run the MSRE-HTPrimer with the test data sets.

Step6: For new data analysis with MSRE-HTPrimer, prepare the Target file and run the primer design.

4. MSRE-HTPrimer web interface description

4.1 MSRE-HTPrimer query page:

User can run primer design with MSRE-HTPrimer by providing input options and files from query page as shown in Figure 1.

Following input parameters and input files are required.

Input 1:

Genome information parameters are required to download genome fasta sequence and annotation files (RefSeq gene, common SNPs, CpG island and known repeat elements) from UCSC genome browser (http://genome.ucsc.edu/index.html)

1) Select genome name from first drop down menu (Human or Mouse). The default genome is Human.
2) Select genome assembly version from second drop down menu (default genome assembly is hg19)
3) Select the dbSNP build to download the corresponding common SNPs from UCSC genome browser. Default is 142 for human, genome assembly hg19.

Input 2:

Upload a target file in BED format. One file for each target region. This file consists of 4 columns 1) chromosome, 2) start position, 3) end position and 4) target ID. User can give any number of target region in a single run.

Input 3:

Third input is the Primer3 input parameters for primer design. This file can be modified as per the requirement and upload. This is an optional input, if user does not provide then MSRE-HTPrimer uses the default setting of Primer3.

Input 4:

This input is only required fo MSRE-PCR primer design, user can either enter type-II enzyme is the input box (one enzyme per line) or alternatively can upload a text file which contains one enzyme per line.

Input 5:

To provide flexibility in primer design process, MSRE-HTPrimer provides some useful input options for optimized and speific primer design and selection. Under these parameters user can define

1. Maximum primer pairs to return for each target region. Default 10

2. Product size: Minimum, Optimum and Maximum. Default 150, 250 and 320 respectively.
3. Primer annealing temperature. Default 52, 60 and 65 for Minimum, Optimum and Maximum temperature respectively.
4. Primer size: Minimum, Optimum and Maximum. Default 22, 28 and 36 bp respectively.

Input 6:

This is unique feature of MSRE-HTPrimer to provide primer selection quality matrix for final primer pair selection, which helps to reduce the post selection process. User can define various filtering criteria for each output column of the MSRE-HTPrimer and then tool automatically selection primers from the whole output. This is optional input.

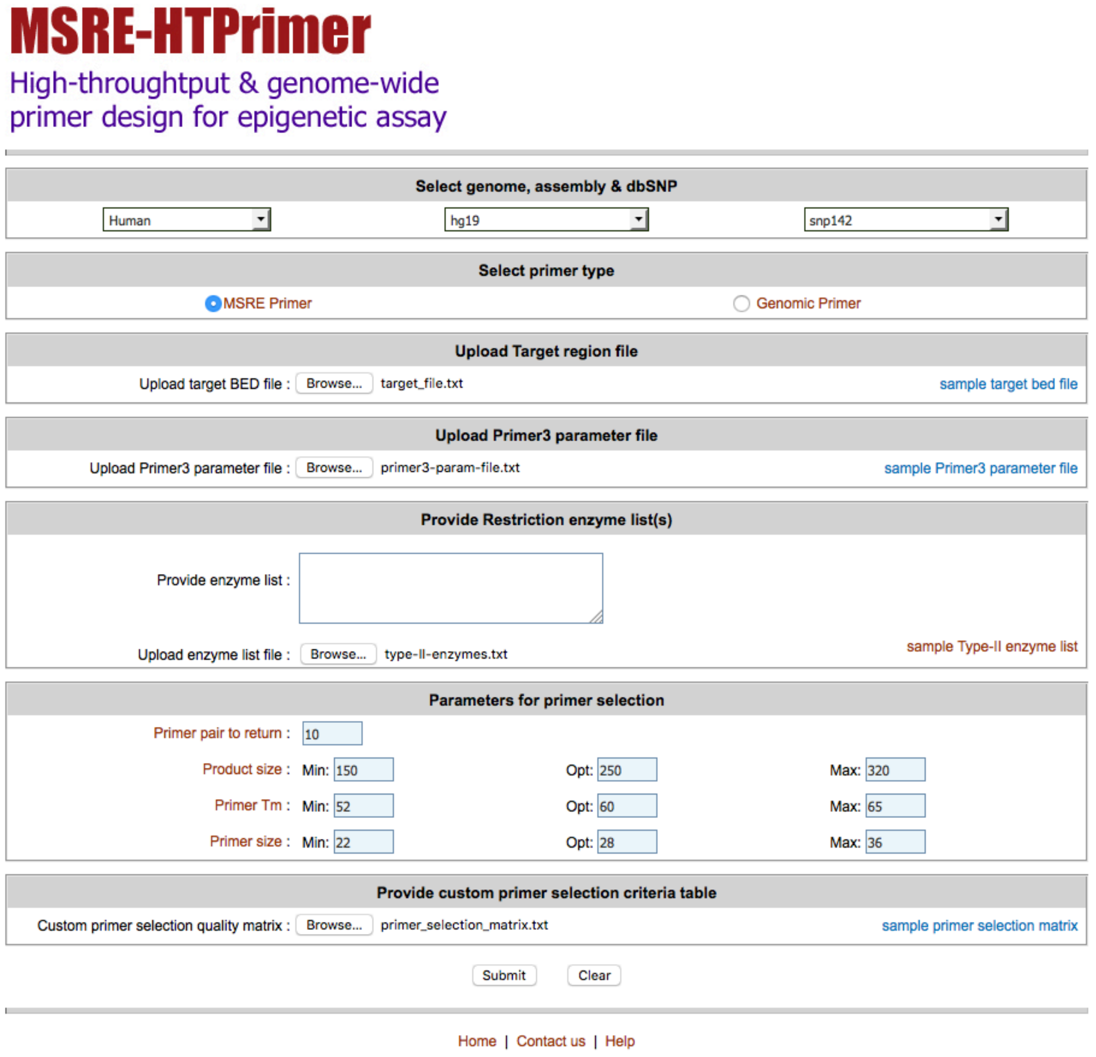

Figure 1: shows MSRE-HTPrimer query page to define all parameters and upload input files for primer design

4.2 MSRE-HTPrimer result page

MSRE-HTPrimer results all primer pairs in a summary table, which is available in HTML to display (as shown in Figure 2) and in TEXT and HTML format to download. Moreover, MSRE-HTPrimer has seamlessly integrated the UCSC genome browser and UCSC In-Silico PCR visualization in the result page. All resulting primer pairs of a target region are visualized in UCSC genome browser as shown in Figure 3. For each primer pair In-Silico PCR is displayed within the result page of MSRE-HTPrimer as shown Figure 4.

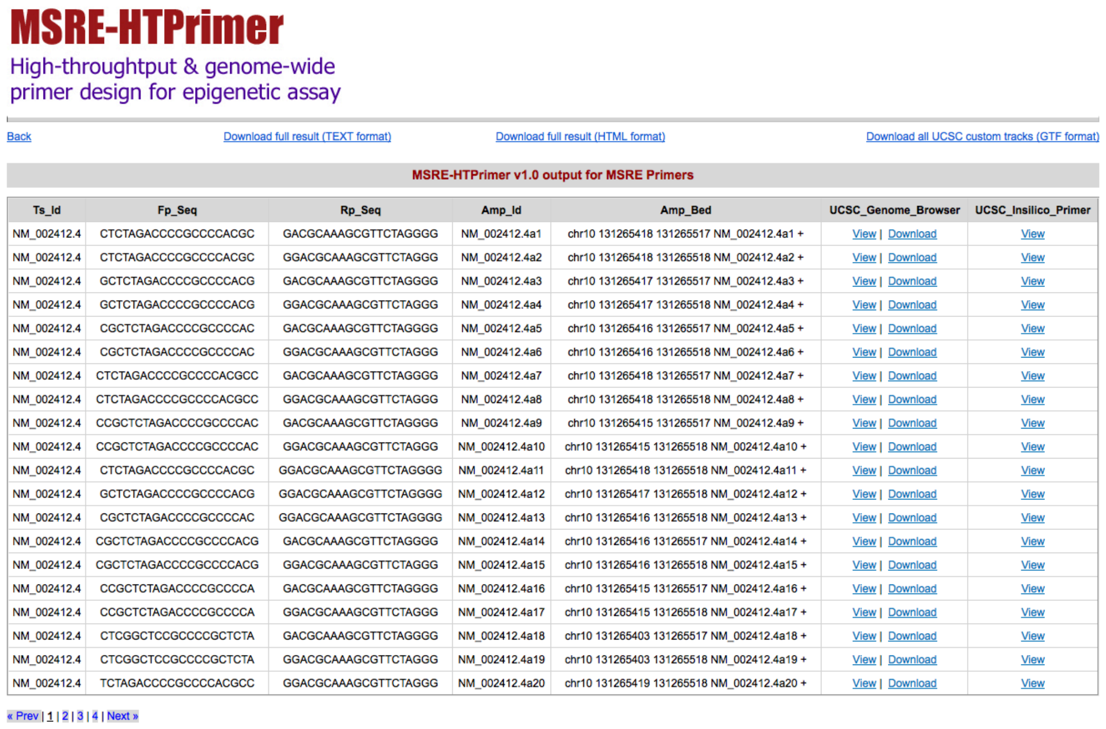
MSRE-HTPrimer
High-throughtput & genome-wide
primer design for epigenetic assay

Back | Download full result (TEXT format) | Download full result (HTML format) | Download all UCSC custom tracks (GTF format)

MSRE-HTPrimer v1.0 output for MSRE Primers

Ts_Id	Fp_Seq	Rp_Seq	Amp_Id	Amp_Bed	UCSC_Genome_Browser	UCSC_Insilico_Primer
NM_002412.4	CTCTAGACCCCGCCCCACGC	GACGCAAAGCGTTCTAGGGG	NM_002412.4a1	chr10 131265418 131265517 NM_002412.4a1 +	View | Download	View
NM_002412.4	CTCTAGACCCCGCCCCACGC	GGACGCAAAGCGTTCTAGGG	NM_002412.4a2	chr10 131265418 131265518 NM_002412.4a2 +	View | Download	View
NM_002412.4	GCTCTAGACCCCGCCCCACG	GACGCAAAGCGTTCTAGGGG	NM_002412.4a3	chr10 131265417 131265517 NM_002412.4a3 +	View | Download	View
NM_002412.4	GCTCTAGACCCCGCCCCACG	GGACGCAAAGCGTTCTAGGG	NM_002412.4a4	chr10 131265417 131265518 NM_002412.4a4 +	View | Download	View
NM_002412.4	CGCTCTAGACCCCGCCCCAC	GACGCAAAGCGTTCTAGGGG	NM_002412.4a5	chr10 131265416 131265517 NM_002412.4a5 +	View | Download	View
NM_002412.4	CGCTCTAGACCCCGCCCCAC	GGACGCAAAGCGTTCTAGGG	NM_002412.4a6	chr10 131265416 131265518 NM_002412.4a6 +	View | Download	View
NM_002412.4	CTCTAGACCCCGCCCCACGCC	GACGCAAAGCGTTCTAGGGG	NM_002412.4a7	chr10 131265418 131265517 NM_002412.4a7 +	View | Download	View
NM_002412.4	CTCTAGACCCCGCCCCACGCC	GGACGCAAAGCGTTCTAGGG	NM_002412.4a8	chr10 131265418 131265518 NM_002412.4a8 +	View | Download	View
NM_002412.4	CCGCTCTAGACCCCGCCCCAC	GACGCAAAGCGTTCTAGGGG	NM_002412.4a9	chr10 131265415 131265517 NM_002412.4a9 +	View | Download	View
NM_002412.4	CCGCTCTAGACCCCGCCCCAC	GGACGCAAAGCGTTCTAGGG	NM_002412.4a10	chr10 131265415 131265518 NM_002412.4a10 +	View | Download	View
NM_002412.4	CTCTAGACCCCGCCCCACGC	GGACGCAAAGCGTTCTAGGGG	NM_002412.4a11	chr10 131265418 131265518 NM_002412.4a11 +	View | Download	View
NM_002412.4	GCTCTAGACCCCGCCCCACG	GGACGCAAAGCGTTCTAGGGG	NM_002412.4a12	chr10 131265417 131265518 NM_002412.4a12 +	View | Download	View
NM_002412.4	CGCTCTAGACCCCGCCCCAC	GGACGCAAAGCGTTCTAGGGG	NM_002412.4a13	chr10 131265416 131265518 NM_002412.4a13 +	View | Download	View
NM_002412.4	CGCTCTAGACCCCGCCCCACG	GACGCAAAGCGTTCTAGGGG	NM_002412.4a14	chr10 131265416 131265517 NM_002412.4a14 +	View | Download	View
NM_002412.4	CGCTCTAGACCCCGCCCCACG	GGACGCAAAGCGTTCTAGGG	NM_002412.4a15	chr10 131265416 131265518 NM_002412.4a15 +	View | Download	View
NM_002412.4	CCGCTCTAGACCCCGCCCCA	GACGCAAAGCGTTCTAGGGG	NM_002412.4a16	chr10 131265415 131265517 NM_002412.4a16 +	View | Download	View
NM_002412.4	CCGCTCTAGACCCCGCCCCA	GGACGCAAAGCGTTCTAGGG	NM_002412.4a17	chr10 131265415 131265518 NM_002412.4a17 +	View | Download	View
NM_002412.4	CTCGGCTCCGCCCCGCTCTA	GACGCAAAGCGTTCTAGGGG	NM_002412.4a18	chr10 131265403 131265517 NM_002412.4a18 +	View | Download	View
NM_002412.4	CTCGGCTCCGCCCCGCTCTA	GGACGCAAAGCGTTCTAGGG	NM_002412.4a19	chr10 131265403 131265518 NM_002412.4a19 +	View | Download	View
NM_002412.4	TCTAGACCCCGCCCCACGCC	GGACGCAAAGCGTTCTAGGG	NM_002412.4a20	chr10 131265419 131265518 NM_002412.4a20 +	View | Download	View

« Prev | 1 | 2 | 3 | 4 | Next »

Figure 2: shows MSRE-HTPrimer primer pair output summary table (http://localhost/msre-htprimer).

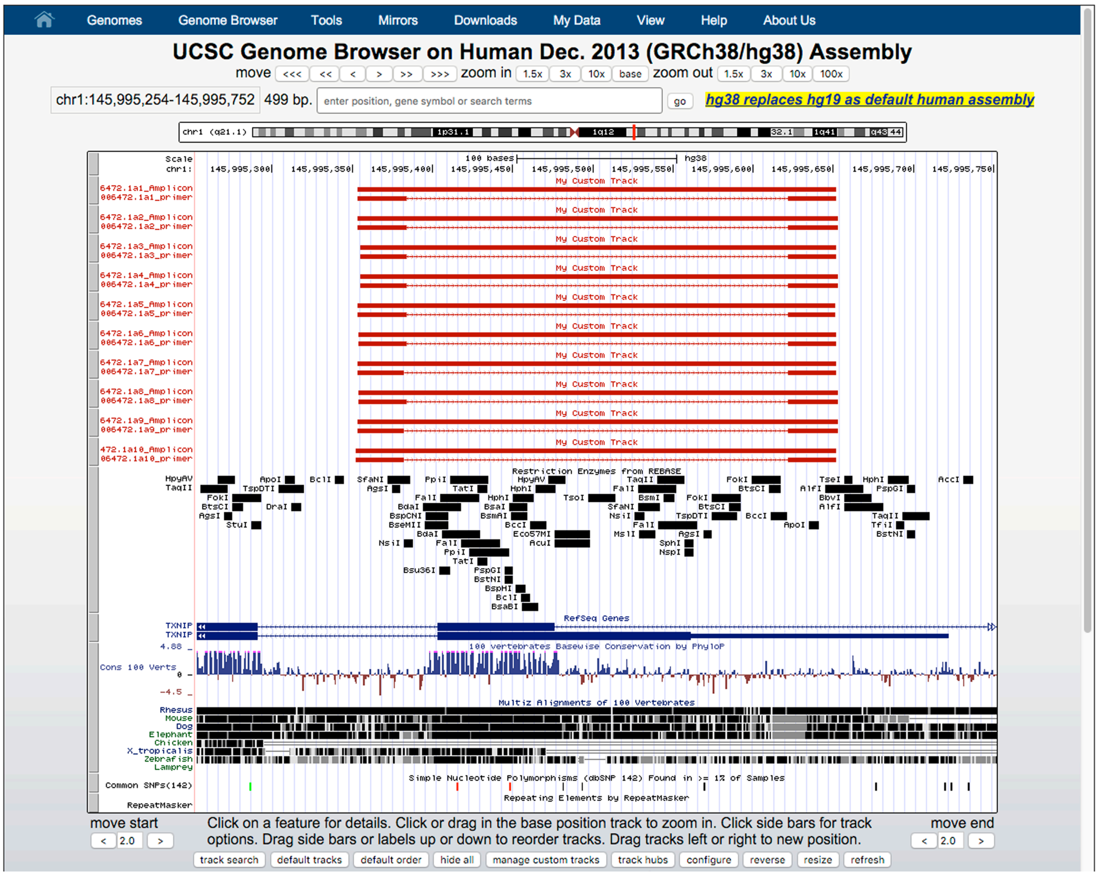

Figure 3: shows the visualization of primer pairs in the UCSC genome browser within the MSRE-HTPrimer result page (http://genome.ucsc.edu/cgi-bin/hgTracks?db=hg38).

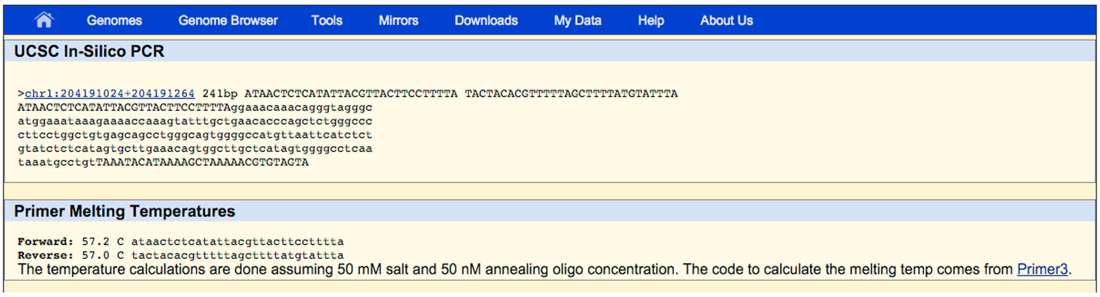

Figure 4: shows the visualization of primer pairs in the UCSC In-Silico PCR within the MSRE-HTPrimer result page (http://genome.ucsc.edu/cgi-bin/hgPcr?org=Human&db=hg38&wp_target=genome&wp_f=AAGATATTTTAGACAGAAAAGTTCAAAGAT&wp_r=AAAATACATCAGAATTTCTCTTTAAGAAC&wp_size=320&wp_perfect=15&wp_good=15&boolshad.wp_flipReverse=0).

4.3 Run MSRE-HTprimer with test input

To validate the installation of the MSRE-HTPrimer pipeline it can be run with a small test data set. The test data set for MSRE-PCR and Genomic-PCR can be obtained from https://sourceforge.net/projects/msrehtprimer/files/test_data.zip and run the following command to uncompress the file:

unzip test_data.zip

Note that after uncompressing the **.zip** file, a new folder will be created named ***test_data***. Now upload these files on the MSRE-HTPrimer query page (http://localhost/msre-htprimer) and run the primer design.

5. MSRE-HTPrimer inputs description

MSRE-HTPrimer requires four input files:

1. Target BED file: This file contains the genomic coordinates for all target sequences (one line for each target sequence). It consists of four tab-delimited columns: 1) chromosome, 2) start coordinate, 2) end coordinate and 4) a unique ID for each target region (S1 Table).

chr2	241454334	241457334	Target1
chr3	10155818	10158818	Target2
chr5	118813546	118816546	Target3
chr5	148183848	148186848	Target4
chr5	112098954	112101954	Target5
chr15	89059082	89062082	Target6
chr19	1154297	1157297	Target7
chrY	25386895	25389895	Target8

2. Primer3 parameter file: This text file contains the parameters and values for the Primer3 tool. It is optional and if not provided, MSRE-HTPrimer will use default Primer3 parameters as shown in figure 5

```
PRIMER_TASK=generic
PRIMER_MISPRIMING_LIBRARY=
PRIMER_MIN_TM=65.0
PRIMER_OPT_TM=70.0
PRIMER_MAX_TM=75.0
PRIMER_MIN_GC=20.0
PRIMER_MAX_GC=100.0
PRIMER_NUM_RETURN=5000
PRIMER_MIN_SIZE=16
PRIMER_OPT_SIZE=21
PRIMER_MAX_SIZE=30
PRIMER_PRODUCT_SIZE_RANGE=50-150
SEQUENCE_ID=TS001
SEQUENCE_TEMPLATE=
PRIMER_PICK_LEFT_PRIMER=1
PRIMER_PICK_RIGHT_PRIMER=1
PRIMER_PICK_INTERNAL_OLIGO=1
PRIMER_PICK_ANYWAY=1
PRIMER_THERMODYNAMIC_OLIGO_ALIGNMENT=0
PRIMER_THERMODYNAMIC_TEMPLATE_ALIGNMENT=0
```

Figure 5: Primer3 parameter file

3. Restriction enzyme file: This input file is only required for MSRE-PCR primer design. Each line contains an enzyme name as per nomenclature and multiple enzymes are allowed in a single run as shown in figure 6.

```
HpaII
Hin6I
AciI
HpyCH4IV
```

Figure 6: Enzyme list in a text file

4. Custom primer selection quality matrix

MSRE-HTPrimer supports further selection of primer pairs based on user defined selection criteria. A custom quality-filtering matrix can be provided as input file. As shown in Table 1, the user can define a set of selection criteria and rank them using a scale of 1-10. MSRE-HTPrimer assigns these ranks to the primer pairs for all target sequences. If this input is not provided then primer pairs are returned based on Primer3 ranking. MSRE-HTPrimer supports mathematical operators, including “>”, “<”, “>=”, “<=” and “-“. Any column header of the MSRE-HTPrimer output file can be used as parameter. The primer quality level represents the rank associated with each of the output parameters in its respective row.

Table 1: Custom quality filter matrix with ten quality levels ranking the designed primer independent of the primer3 level, but dependent on amplicon size, amount of cutsites and gene distance.

Primer_Quality_Level	Fp_Tm	Fp_Gc_%	Fp_Any_Compl	Rp_3'_Compl	Amp_Size	Lp_Repeat_In_Bp	Lp_Snp_Pos_From_3'	Rp_Repeat_In_Bp	Rp_Snp_Pos_From_3'	Hyb_Repeat_In_Bp	Hyb_Snp_Pos_From_3'	Amp_Sum_Cutsites_Primer	Amp_Sum_Cutsites_Between_Primers
1	65-75	30-70	0-2.55	0-2.55	90-110	0	>10	0	>10	>3	>10	0	>4
2	65-75	30-70	0-2.55	0-2.55	90-120	0	>10	<2	>10	>3	>10	>=3	>3

3	65-75	30-70	0-2.55	0-2.55	80-140	<5	>10	<3	>10	>4	>10	>=3	>2
4	65-75	30-70	0-2.55	0-2.55	80-140	<=5	>10	<4	>10	>5	>10	>=3	>2
5	65-75	30-70	0-2.55	0-2.55	80-140	<5	>10	<5	>10	>6	>10	>=3	>2
6	65-75	30-70	0-2.55	0-2.55	80-140	<10	>10	<=6	>10	>7	>10	>=3	>1
7	65-75	30-70	0-2.55	0-2.55	80-140	<10	>10	<7	>10	>8	>10	>=3	>1
8	65-75	30-70	0-2.55	0-2.55	80-140	<10	>10	<8	>10	>9	>10	>=3	>1
9	65-75	30-70	0-2.55	0-2.55	80-140	<10	>10	<10	>10	>10	>10	>=3	>1
10	65-75	30-70	0-2.55	0-2.55	80-140	<10	>5	>9	>5	>11	>=5	>=3	>0

All headers consist of two major parts, origin (Fp=forward primer, Lp=left primer, Rp=reverse primer/right primer, Amp=amplicon, Hyb=hybridization oligo) and short description. Primer_Quality_Level=user defined rank; Tm=melting temperature of origin; Gc_%=GC percentage in DNA sequence of origin; Any_Compl=stability of any basepairing of origin to itself; 3'_Compl= stability of any basepairing of the 3' end of the origin to itself; Size=size of origin in basepairs (Bp); Repeat_In_Bp=allowed Bp of repeats in origin; Snp_Pos_From_3'=distance of closest SNP position to 3’end inside the origin sequence in basepairs; Amp_Sum_Cutsites_Primer=amount of cutsites in FP and RP; Amp_Sum_Cutsites_Between_Primers=amount of cutsites in amplicon except for FP and RP.

6. How to use MSRE-HTPrimer?

To use MSRE-HTPrimer for primer design user

Open web browser and type the following URL into browser (http://localhost/msre-htprimer) and upload required inputs files, change default parameters if required and run the primer design.

7. Contact Information

PD Dr. Andreas Weinhäusel
andreas.weinhaeusel@ait.ac.at
Ram Vinay Pandey
ramvinay.pandey@gmail.com
